# 1D micro-nanopatterned integrin ligand surfaces for directed cell movement

**DOI:** 10.3389/fcell.2022.972624

**Published:** 2022-12-02

**Authors:** Victoria Levario-Diaz, Rebecca Elizabeth Alvarado, Cristina Marcela Rodriguez-Quinteros, Andreas Fink, Joel Christian, Wenqian Feng, Elisabetta Ada Cavalcanti-Adam

**Affiliations:** ^1^ Department of Cellular Biophysics, Max Planck Institute for Medical Research, Heidelberg, Germany; ^2^ College of Polymer Science and Engineering, Sichuan University, Chengdu, China

**Keywords:** nanotechnology/nanomaterials, micropatterning of surface, integrins, focal adhesion (FA), cell adhesion

## Abstract

Cell-extracellular matrix (ECM) adhesion mediated by integrins is a highly regulated process involved in many vital cellular functions such as motility, proliferation and survival. However, the influence of lateral integrin clustering in the coordination of cell front and rear dynamics during cell migration remains unresolved. For this purpose, we describe a novel protocol to fabricate 1D micro-nanopatterned stripes by integrating the block copolymer micelle nanolithography (BCMNL) technique and maskless near UV lithography-based photopatterning. The photopatterned 10 μm-wide stripes consist of a quasi-perfect hexagonal arrangement of gold nanoparticles, decorated with the RGD (arginine-glycine-aspartate) motif for single integrin heterodimer binding, and placed at a distance of 50, 80, and 100 nm to regulate integrin clustering and focal adhesion dynamics. By employing time-lapse microscopy and immunostaining, we show that the displacement and speed of fibroblasts changes according to the nanoscale spacing of adhesion sites. We found that as the lateral spacing of adhesive peptides increased, fibroblast morphology was more elongated. This was accompanied by a decreased formation of mature focal adhesions and stress fibers, which increased cell displacement and speed. These results provide new insights into the migratory behavior of fibroblasts in 1D environments and our protocol offers a new platform to design and manufacture confined environments in 1D for integrin-mediated cell adhesion.

## Introduction

Directed cell migration is a key event in physiological and pathological processes, such as embryonic morphogenesis, tissue formation, wound healing and cancer metastasis. In both single and collective cell migration, a continuous front-rear coordination is required for cell adhesion dynamics, where extracellular chemo-mechanical cues guide cell polarization and migration behavior (Ladoux et al., 2016). Cell adhesion to the extracellular matrix (ECM) is mediated by integrins, transmembrane receptors linking the ECM to the cytoskeleton. Upon binding to ECM ligands, multiple intracellular adaptor proteins, such as talin, paxillin and vinculin, are recruited, leading to integrin clustering and formation of focal adhesions (FAs) (Critchley, 2000). These complex and dynamic assemblies provide the anchor points necessary to generate cellular tension, symmetry breaking and traction force to initiate cell motility (Balaban et al., 2001; Nagano et al., 2012). Several studies provided evidence regarding the importance of extracellular environment heterogeneity and chemical gradients for cell migration plasticity (Friedl and Wolf, 2010; Tozluoglu et al., 2015; Talkenberger et al., 2017). During migration, the molecular dynamics at the front and rear of single cells, such as mesenchymal fibroblasts, are driven by protrusions at the front and retraction at the rear establishing a polarity in response to external biochemical and physical cues. For example, haptotaxis is a type of cell motility based on the presence of density gradients provided by the ECM that is strongly dependent on adhesion strength and results in cell contractility (Wen et al., 2015). Previous work in our group demonstrated by using nanoscale RGD ligand haptotactic gradients that cells orient towards the smallest ligand spacing and can detect differences in spacings as low as 1 nm across their body (Arnold et al., 2008; Hirschfeld-Warneken et al., 2008). Even in the absence of external cues to initiate cell polarization, few studies have proposed spontaneous signals that cells could self-generate such as external chemoattractant gradients (Tweedy et al., 2016) or intrinsic actin cytoskeleton pattern transitions (Tee et al., 2015) to become migratory.

Micropatterned surfaces have become a valuable and essential tool for manipulating cell adhesion in defined regions and investigating the impact of microenvironments on cell physiology (Thery, 2010). Current techniques for obtaining micropatterned surfaces are based on photolithographic processes and can range from direct writing techniques, such as molecular and protein printing (Scott et al., 2012), to indirect techniques such as soft lithography (Qin et al., 2010) and photolithography (D'Arcangelo and McGuigan, 2015). For indirect techniques, elastomer stamps or photomasks have been widely used to create microscale patterns. However, issues on compatibility and alignment of prefabricated microstructures arise in wet environments where biomolecules are present. To overcome these problems, maskless projection lithography systems based on digital micromirror devices (DMD) have recently been developed (Strale et al., 2016). The predesigned pattern is projected and exposed on a photosensitive layer, requiring a few mWmm^−2^ of UV light and reaching a micrometric resolution on the substrate (Zhao et al., 2009). Furthermore, the development of 2D surfaces that allow controlled cell adhesion with a precise spatio-temporal presentation of ligands, such as RGD motifs, at a nanometer scale has been increasing in recent decades. Metal-based nanopatterning techniques using gold (Au) or titanium dioxide (TiO_2_) in combination with self-assembly methods such as colloidal lithography, and nanolithography have expanded the geometry (Schvartzman et al., 2011) and topography (Dalby et al., 2004) of adhesion sites reaching single molecule-level control (Jain et al., 2022). Among the first self-assembly techniques reported is the fabrication of nanoengineered surfaces with RGD-bound gold nanoparticles based on diblock copolymer micellar nanolithography (BCMNL). Here, the nanoscale spacing of integrin ligands results in the regulation of integrin lateral clustering, which affects mechanosensing and generation of molecular and cellular forces at focal adhesions (Spatz et al., 2000; Arnold et al., 2004; Cavalcanti-Adam et al., 2007; Liu et al., 2014; Oria et al., 2017). These observations have also been reported using different self-assembly systems, such as polystyrene-block-ethylene oxide maleimide (PS-PEO-Ma), where the increase in the ligand lateral spacing affects the migratory behavior and differentiation of human mesenchymal stem cells (hMSCs) (Frith et al., 2012). While these previous studies elucidated the spatial density of ECM ligands required to sense the environment and initiate 2D cell migration, a better understanding of front-rear coordination in response to ligand spacings can be achieved by fabricating confined geometries. Cells migrating in uniform 1D adhesive tracks have been reported to exhibit an initial non-migratory phase by remaining in a symmetrically elongated shape. Subsequently, a spontaneous symmetry break occurs characterized by a protrusion at the cell front and a rapid retraction at the rear, generating a stick-slip migration mode (Ziebert and Aranson, 2013; Sens, 2020). This transition from a steady motion to a stick-slip mode can occur sporadically and be persistent, changing the direction of migration (Ron et al., 2020). Since integrins are responsible for coupling and maintaining the cytoskeleton-substrate adhesion, it remains unknown whether the clustering of integrins is involved in the regulation of cell movement and stabilization of the cell body for efficient nuclear translocation and retraction in directed cell migration.

To address these questions, we developed a combined micro-nanopatterning setup to control integrin clustering in cells migrating in 1D microenvironments. This method consists in the manufacture of 10 µm wide adhesive stripes by combining the BCMNL technique and maskless UV photopatterning. Gold nanoparticles with interparticle distance of 50–80–100 nm biofunctionalized with RGD peptides served as anchor points to carry out integrin-mediated cell adhesion. Compared to earlier work by our group in which adhesive square patches were created by conventional e-beam lithography on nanostructured surfaces (Arnold et al., 2009), the present protocol provides the opportunity to create adhesive tracks comprising a larger surface area, in addition to reducing manufacturing complexity, cost and time. Our results suggest that integrin clustering during fibroblast movement on 1D micro-nanopatterned stripes participates in the regulation at the front of the cell due to differential activation of the engaged adhesion sites, and in the stabilization of retraction events at the rear. Furthermore, depending on the ECM ligand spacing, different migration modes emerge within a uniform population of cells.

## Materials and methods

### Preparation of nanopatterned surfaces

The method for preparing nanopatterned surfaces based on self-assembled diblock copolymer micelles containing gold nanoparticles has been previously described (Arnold et al., 2004; Cavalcanti-Adam et al., 2007). Briefly, glass coverslips (20 mm × 20 mm, Carl Roth & Co GmbH, Karlsruhe, Germany) were cleaned with a mixture of [3:1 H2SO4/H2O2 (33%)] for approximately 45 min, rinsed extensively with MilliQ water and dried under a stream of nitrogen. Different compositions of polystyrene (PS) (*x*)-*block*-poly (2-vinylpyridine) (*y*) copolymers (PolymerSource Inc.) were used to generate three different spacings of gold nanoparticles (Table 1). The copolymers were dissolved in a 2 mg ml^−1^ extra dry toluene solution and allowed to stir for 24 h at room temperature. Then, HAuCL_4_•3H_2_O (Sigma Aldrich) was added into the micellar solutions with a loading parameter of *L* = 4, defined as $L=n\left[HAu{CL}_{4}\right]/n\left[P2VP\right]$ and left to stir for 24 h. The gold-loaded micellar solutions were filtered and 25 μl was used to cover clean glass coverslips by spin coating (Spin Coater WS 650 Mz-23 MPP, Laurel Technologies Corporation). The coated surfaces were then treated with H_2_ plasma for 5 min (H_2_ pressure: 0.4 mbar, power: 150 W; Diener Atto, plasma cleaner) to remove the polymers and leave the bare gold nanoparticles on the glass surfaces.

**TABLE 1:** Molecular weights of diblock copolymers and gold interparticle spacings.

Polymer^a^ PS(x)-*b*-P2VP(y)	Mn PS (g mol^−1^)	Mn PVP (g mol^−1^)	Spin coating [rpm]	Interparticle distance [nm]^b^
1200-*b*-556	125,000	58,500	2,500	50 ± 13
1200-*b*-556	125,000	58,500	8,000	80 ± 12
1824-*b*-523	190,000	55,000	4,000	100 ± 16

^a^ *x* and *y* are the monomer repeat units for the diblock copolymers.

^b^ Gold interparticle spacing obtained by scanning electron microscopy.

### Micropatterning of nanopatterned surfaces using maskless photolithography

Gold nanoparticle surfaces were oven dried for 20 min at 120°C before coating with a mixture of 1:2 negative photoresist/negative resist thinner (Sigma Aldrich) to create 1D micropatterned stripes using the maskless photolithography technique. The maskless near UV lithography-based photopatterning technique has been previously described (Strale et al., 2016). The photopatterning module uses a near-UV solid-state laser source to create micropatterns on glass surfaces. Briefly, the UV-A laser was calibrated using a ×20 air objective and adjusting the focus on the surface where the nanopattern was located. Surface patterns were drawn using the graphic software Inkscape. Each glass coverslip was patterned covering an 8 mm central region with lines having 10 μm width and 400 μm length, at center-to-center distance of 200 μm from each other. The employed UV dose was 7 mJ/mm^2^ and the duration time of the patterning was approximately 10 min per surface. The micropatterned stripes were developed by spin coating the glass coverslips with xylene and subsequently with isopropanol for 3 min at 8,000 rpm, followed by oven drying at 80°C for 20 min.

To remove the gold nanoparticles not protected by a layer of cured photoresist, surfaces were sonicated for 2 h in a solution of 2 mM cysteamine hydrochloride in MilliQ water with 1 ml isopropyl alcohol to reduce the water surface tension (pH 10, adjusted with 2 M NaOH). Dry ice was constantly added to the sonicator as the temperature increased by the energy emitted and a low-temperature solution was required. Once sonication was completed, glass surfaces were washed with MilliQ water and subsequently spin-coated with acetone and isopropanol for 3 min at 8,000 rpm to remove the photoresist covering the 1D patterned lines containing gold nanoparticles. An additional UV treatment for 10 min was used to further clean the glass surfaces. The surfaces were then treated with H_2_ plasma for 45 min (H_2_ pressure: 0.4 mbar, power: 450 W; Diener Atto, plasma cleaner). The surface between the gold nanoparticles was passivated with methoxy polyethylene glycol silane (mPEG-silane, molecular weight 2 kDa; Biopharma PEG) to prevent unspecific protein adsorption and cell adhesion. After 48 h, the surfaces were incubated for 2 h at room temperature with a thiolated cyclic peptide c (-RGDfE) (Peptide Specialty Laboratories GmbH, Germany) to functionalize the gold nanoparticles.

### Cell culture

REF52 cells (Rat embryonic fibroblast cell line) stably transfected with paxillin fused to yellow fluorescent protein (REF52-YFP-PAX) were a kind gift from Benjamin Geiger and Alexander Bershadsky (the Weizmann Institute of Science, Rehovot, Israel) (Zamir et al., 1999). Cells were cultured in 25 cm^2^ flasks with Dulbecco’s modified Eagle’s medium (DMEM, Gibco Laboratories, Germany) supplemented with 4.5 g/L glucose, 0.58 g/L L-glutamine, 10% foetal bovine serum (FBS, Gibco Laboratories 10500-064) and 1% penicillin/streptomycin (P/S, Gibco Laboratories 15140-122) at 37°C with a 5% CO_2_ humidified atmosphere prior to experiments. For microscopy imaging, patterned surfaces were glued to custom-made Petri dishes with holes and maintained with 1x PBS supplemented with 1% P/S prior to cell seeding. Cells were detached with 0.25% Trypsin-EDTA (1x) (Gibco Laboratories 25200-072) and seeded at a density of 2,000/sample in DMEM supplemented with 1% FBS for all time-lapse imaging experiments.

### Cell movement and nuclei detection

Before seeding REF52-YFP-PAX cells on the patterned surfaces, Silicon rhodamine (SiR)-DNA (Spirochrome) was added to the medium at a concentration of 1:2000 to label cell nuclei. Cells were then allowed to adhere for 2 h before time-lapse imaging. Single cells were visualized and imaged with a DeltaVision system (Applied Precision Inc., Issaquah, WA, United States) on an Olympus IX inverted microscope (Olympus, Hamburg, Germany) employing a 10x/0.40 UplanSApo objective. REF52-YFP-PAX cells were maintained at 37°C in a heated chamber with 5% CO_2_ while time-lapse images were taken every 5 min for 15 h. The movement of individual cells and the detection of their nuclei on surfaces with a linear pattern were taken in polarized (POL) light and with a 632/22 nm laser (Cy5), respectively.

### 1D cell movement and velocity analysis

The cell displacement on patterned lines was analyzed in 1D by tracking the center of the stained nuclei over each frame of the time-lapse movies. To this end a Python script was employed, in which first a threshold filter was applied to the nucleus channel of each image in the time-lapse. This allowed for an automated detection of the nucleus outline and identification of nucleus center. The threshold was chosen manually for each time-lapse video to allow for a reliable detection of the nucleus. Only cell movements along lines were considered by applying principal component analysis to each cell trajectory. For all further analysis the dimensionality of the 2D data was reduced by projecting the cell position onto this first principal component, describing the cell movement in 1D (Rossum and Drake, 2009; Pedregosa F. et al., 2011; Harris et al., 2020; Virtanen et al., 2020). The units of the raw data that were obtained from the time-lapse imaging (i.e., given displacement in pixels) were converted to µm and the time frames to time in h. To calculate the velocity, the displacement in µm between each time frame was divided by 5 min, which is the time elapsed between each frame.

### Immunostaining and focal adhesion analysis

After time-lapse imaging, cell medium was removed from the Petri dish and cells were fixed with 4% paraformaldehyde at room temperature for 15 min. Surfaces were then washed three times with 1x PBS and then permeabilized with 0.1% v/v Triton-X 100 in PBS for 5 min at room temperature. After washing twice with 1x PBS, a blocking solution of 1% BSA (bovine serum albumin) in PBS was added before primary antibody incubation for 1 h to prevent non-specific antibody binding. The primary antibody, mouse monoclonal anti-vinculin (Sigma Aldrich V9131, 1:100) was diluted in a solution of 1% BSA in PBS and incubated in a humidified chamber overnight at 4°C. After primary antibody incubation, surfaces were washed three times with 1% BSA. Secondary antibody (donkey anti-mouse Alexa Fluor 647 (Thermo Scientific A-31571, 1:200) was then diluted in a solution of 1% BSA in PBS and incubated for 1 h protected from the light at room temperature. Phalloidin-TRITC (Sigma Aldrich P1951, 1:200) and DAPI (Sigma Aldrich D9542, 1:1000) to stain F-actin and nuclei, respectively, were also added to the secondary antibody solution. Surfaces were then washed three times with 1% BSA and mounted with Mowiol on microscope slides previously cleaned with 70% EtOH. Immunofluorescence images were acquired on a Zeiss LSM880 confocal microscope using the ×20 dry objective and ×63 oil immersion objective.

Focal adhesion (FA) analysis was performed following a protocol (Horzum et al., 2014) to measure the size of FAs for each cell (*n* = 15 cells per gold nanoparticle spacing) adhered to micropatterned-nanopatterned surfaces. FA measurements were made using a single fluorescence channel (647-vinculin) and processed on a gray scale. The formed FAs were determined as regions of interest (ROIs) and Feret’s diameter was selected from ImageJ software (Research Services Branch, Image Analysis Software Version 1.53f, NIH, Bethesda, MD, United States).

Adhesion dynamics were analysed by time-lapse imaging of REF52-YFP-PAX cells adhered in 1D micro-nanopattern lines. Cells were maintained at 37°C with a 5% CO_2_ humidified atmosphere while time-lapse images were taken every 10 min for 3 h with a DeltaVision system (Applied Precision Inc., Issaquah, WA, United States) on an Olympus IX inverted microscope (Olympus, Hamburg, Germany) employing a ×60 oil immersion objective. Kymographs of adhesion dynamics were plotted for each ligand spacing using ImageJ software (Research Services Branch, Image Analysis Software Version 1.53f, NIH, Bethesda, MD, United States).

### Scanning electron microscopy and critical point drying

Micropatterned surfaces after the final H_2_ plasma treatment were sputter-coated with a ∼8 nm carbon layer prior to SEM imaging. A Zeiss Ultra 55 field-emission scanning electron microscope (FE-SEM, LEO/Zeiss GmbH, Oberkochen, Germany) was used to visualise the micro-nanopatterned stripes with an applied acceleration voltage of 5 kV. The hexagonality and the spacing of the gold nanoparticles were later analysed using ImageJ software (Research Services Branch, Image Analysis Software Version 1.53f, NIH, Bethesda, MD, United States).

REF52-YFP-PAX cells attached to micro-nanopatterned stripes functionalized with the RGD ligand were fixed in 2% glutaraldehyde in PBS (Sigma-Aldrich) for 15 min and dehydrated in graded ethanol (25%–100%). Critical point drying was performed in a CPD 030 critical point dryer (Bal-Tec) replacing the ethanol with liquid CO_2_ at a pressure of 50 bar and a temperature of 15°C which was then phase changed to gaseous CO_2_ at a pressure of 70 bar and a temperature of 40°C. Samples were then coated with ∼8 nm carbon layer in preparation for SEM imaging. For these samples, an accelerating voltage of 3 kV was applied and adherent cells were analyzed using ImageJ software (Research Services Branch, Image Analysis Software Version 1.53f, NIH, Bethesda, MD, United States).

### Statistical analysis

Statistical analysis was performed for 50 cells per gold nanoparticle spacing for single cell analysis. Data represent mean values with their respective standard deviations (SD) calculated from independent experiments. Statistical analysis was performed in GraphPad Prism (GraphPad Software, San Diego, CA, United States) and significance between data was tested when we performed a one-way ANOVA test with a post hoc Tukey’s multiple comparison test to compare the means of three independent groups. The results were considered statistically significant, *p <* 0.05.

## Results

**FIGURE 1:**
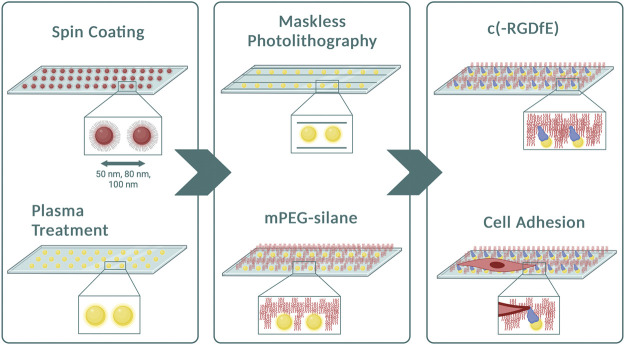
Preparation of micropatterned 1D-RGD-nanopatterned surfaces. Diagram of the method illustrating the main steps to obtain 1D micro-nanopatterned striped surfaces. After spin-coating the micellar solution containing gold nanoparticles to create different particle spacings, plasma treatment was performed to remove the polymers from the surfaces. Using the maskless photolithography technique, lines with a width of 10 μm were patterned. The surfaces were then covered with mPEG-silane to prevent non-specific protein adsorption prior to conjugating naked gold nanoparticles with the c (-RGDfE) peptide to regulate the integrin-mediated fibroblast adhesion. Created with Biorender.com.

**FIGURE 2:**
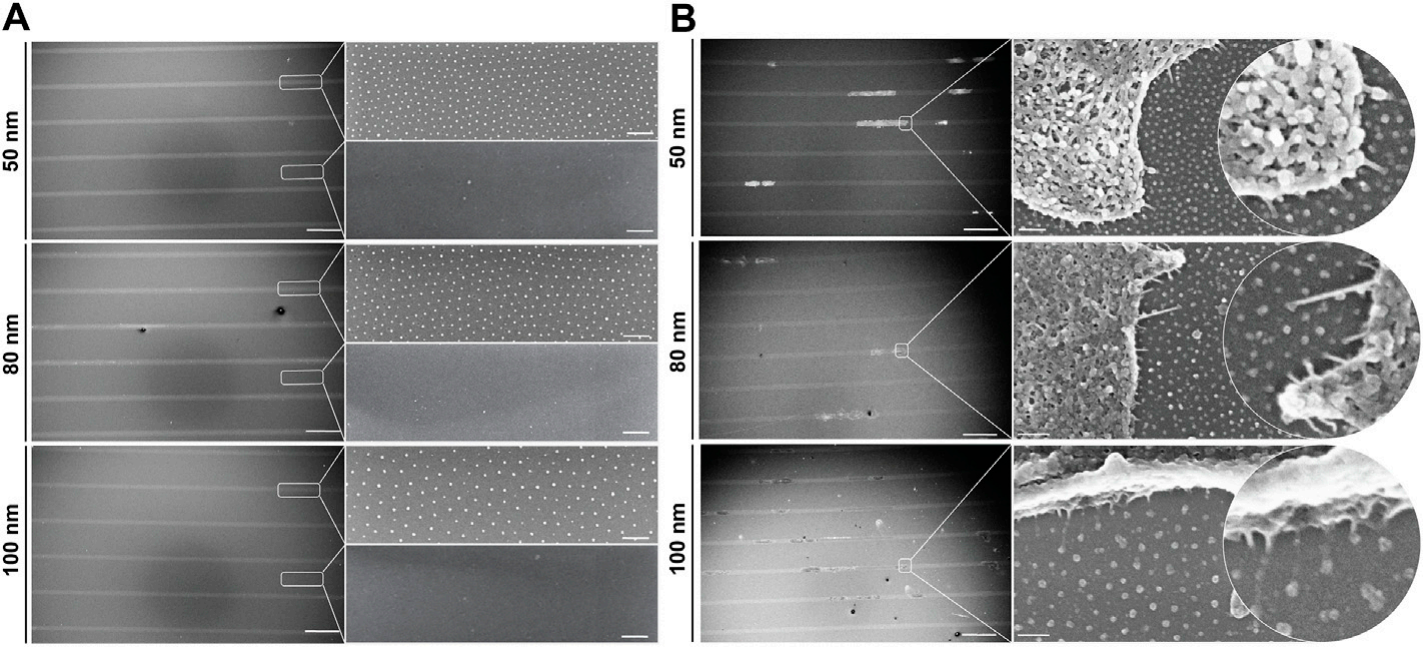
1D micro-nanopatterned surfaces based on the BCMNL technique. Scanning electron microscope images of **(A)** nanopatterned surfaces with 10 μm micropatterned lines showing the quasi-hexagonal arrangement of gold nanoparticles within the patterned line and the absence of gold particles outside the line after being removed by cys hydrochloride solution. **(B)** Critical point drying of cells adhered to gold nanoparticles functionalized with c (-RGDfE) in the 1D micropatterned lines for all gold nanoparticle spacings (50, 80 and 100 nm). Scale bars patterned lines, 200 μm; gold nanopatterns (inserts), 200 nm.

To investigate the dynamics of single cells adhered to nanopatterned surfaces and determine whether the distance between RGD-functionalized gold nanoparticles influences directed cell migration, we developed a bottom-up approach combining two well-established techniques: block copolymer micelle nanolithography (BCMNL) and maskless near-UV lithography patterning (Figure 1). The fabrication steps to obtain 1D micro-nanopatterned integrin ligand surfaces are fully described in *Materials and Methods*. The end product of the fabrication of 10 µm wide photopatterned lines with the quasi-perfect hexagonal array of gold nanoparticles were confirmed by scanning electron microscopy (SEM) images (Figure 2A), while the interparticle spacing was analyzed with ImageJ (Table 1).

**FIGURE 3:**
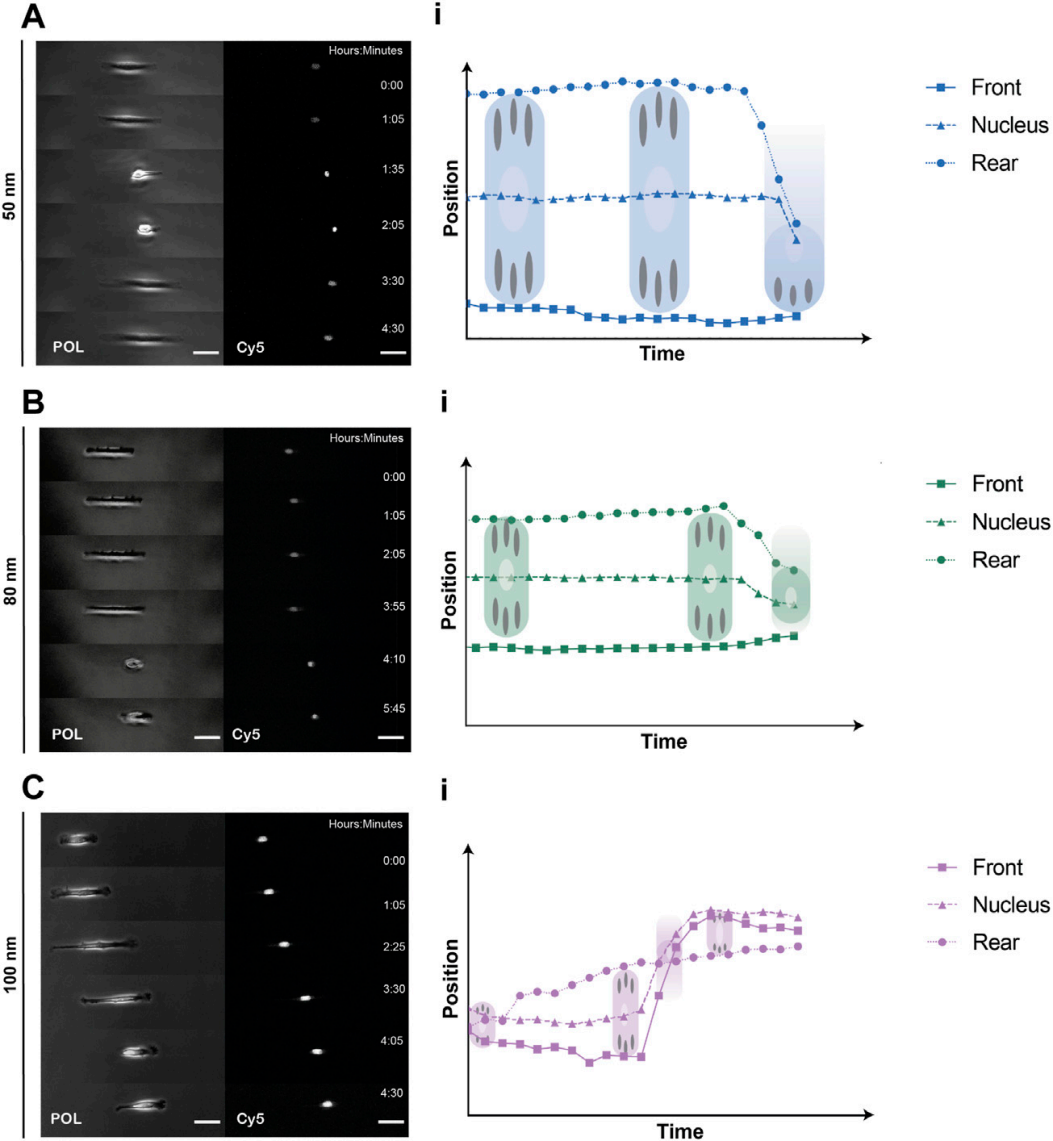
Representative REF52-YFP-PAX cells migrating on 1D micro-nanopatterned surfaces. Polarized (POL) and nuclei (Cy5) images of migrating REF-YFP-PAX cells during the first 8 h for **(A)** 50 nm, **(B)** 80 nm and **(C)** 100 nm gold nanoparticle spacing. Schematic representation of front, rear, and nuclei tracking positions over time during the spreading and retraction phases showing the rear retraction and nuclei translocation for **(Ai)** 50 nm, **(Bi)** 80 nm, and **(Ci)** 100 nm gold nanoparticle spacing. Scale bars, 50 μm.

To quantitively analyze cell displacement on the 1D micro-nanopatterned stripes, REF52-YFP-PAX cells were labeled with a nucleus tracking dye and seeded on the substrates for 2 h prior to time-lapse imaging. Front and rear positions were evaluated by imaging the cells under polarized light, while labeled nuclei were tracked by imaging the emitted fluorescent light (Figures 3A–C, Supplementary Videos S1–S3). From the obtained cell trajectories under polarized light, it was possible to distinguish a generalized migratory behavior for all ligand spacings characterized by an 1) initial symmetric distribution at the front and back of the cell with the nucleus in the center, 2) the extension of the lamellipodia at one end of the cell and, 3) the beginning of directed migration followed by a rapid retraction of the rear and nucleus translocation. This stochastic migration behavior is explained by the stick-slip mechanism (Hennig et al., 2020; Sens, 2020). Notably, we could further observe in the absence of external cues that in the initial spreading phase along the same axis of the linear pattern, the cells symmetrically extended exhibiting two protrusions and strong adhesions to the substrate at both ends, behaving as a force dipoles (Mandal et al., 2014). Then, the cell reached a maximum length without being able to sustain adhesion to the substrate, causing a redistribution of adhesions at the leading edge. Finally, the rear end lost the adherence to the substrate, causing its rapid retraction and translocation of the nucleus. Moreover, the cells adhered on 1D micro-nanopatterned lines exhibited differences in cell morphology. Cell elongation increased along with increasing ligand spacing. In adhesive lines with ligand spacings of 50 and 80 nm, cells were found to be symmetrically elongated, showing greater adhesive interaction with the micro-nano environment while occupying the total width of the photopatterned line. While in the cells adhered at 100 nm spacing, both cell edges occupied the thickness of the line extending the cell mid-body until trailing edge detachment (Supplementary Videos S1–S3). The differences in cell elongations were accompanied by different cell displacements for the three ligand spacings.

**FIGURE 4:**
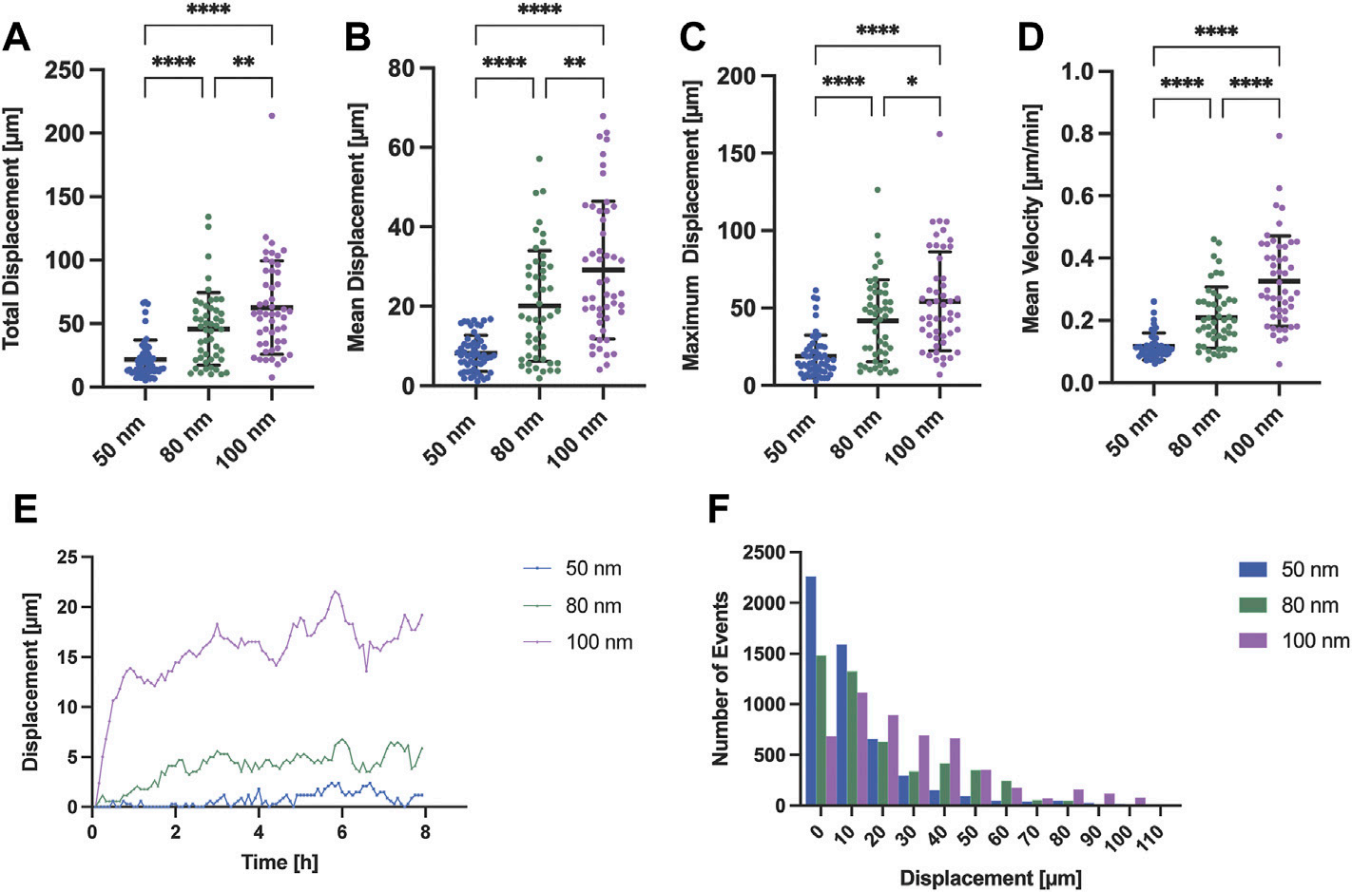
Migration analysis extracting the nuclei position of REF52-YFP-PAX cells adhered to surfaces with different gold interparticle spacing. Scatter plots with means and SD comparing **(A)** the total displacement, **(B)** the mean displacement, **(C)** the maximum displacement and **(D)** mean velocity of migrating cells for all gold nanoparticle spacings (50, 80 and 100 nm; *n* = 50 cells per gold nanoparticle spacing). **(E)** Time profile-median displacements and **(F)** histogram representing the number of events for each distance traveled by all cells (*n* = 50 cells per gold nanoparticle spacing; total *n* = 150 cells) extracted from 8 h of time-lapse imaging are also shown. Statistical significance of three independent experiments was tested with a one-way ANOVA test with a post hoc Tukey’s multiple comparison test. ****p < 0.0001, **p < 0.01, *p = 0.035.

To corroborate what was observed under polarized light, we tracked and extracted the positions of the stained nuclei as a measure of protrusion and retraction cycles that the cells experienced during movement. Cells generally exhibited a tendency to increase displacement and speed when adhered to substrates with nanoparticles spaced further apart (Figures 4A–D). For the case of nanoparticles separated by 50 nm, the cells mostly remained in the spreading phase, exhibiting a smaller displacement (Figure 4F) and, therefore, a lower speed compared to larger gold nanoparticle spacings. This propagation efficiency was previously reported with a ligand spacing of 60 nm or less in ligand cluster arrays with at least four adhesion sites (Schvartzman et al., 2011). In contrast, cells adhered to the nanoparticles separated by 80 and 100 nm were subjected to repeated cycles of protrusion and retraction showing a greater variability in their distribution, both in displacement and speed. Interestingly, it can be seen that the change in the average displacement of cells occurs during the first half hour (Figure 4E) with the average velocity increase of 0.1 μm/min for approximately every 20 nm gold nanoparticle spacing difference (Figure 4D). These results are in agreement with previous work where it was shown that cells sense RGD-nanopatterned surfaces *via* thin membrane projections, filopodia, similar as shown in Figure 2B. On 2D surfaces with spacings of 58 nm and 108 nm, filopodia experienced local retractions and their length increased according to the spacing of RGD-bound gold nanoparticles (Cavalcanti-Adam et al., 2007). However, previous studies using two different self-assembly systems (BCMNL and PS-PEO-Ma) also reported that migration rate of mesenchymal cells on 2D surfaces decreases with increasing lateral ligand spacing (Cavalcanti-Adam et al., 2007; Frith et al., 2012). The migratory behavior described in 2D surfaces is clearly the opposite from our 1D adhesive line results where the rate increases along with the lateral ligand spacing. Considering the morphological observations and the differences in the protrusion-retraction cycles, we reasoned that the migration rate might depend on the adhesion strength which is regulated by the lateral spacing of peptides.

**FIGURE 5:**
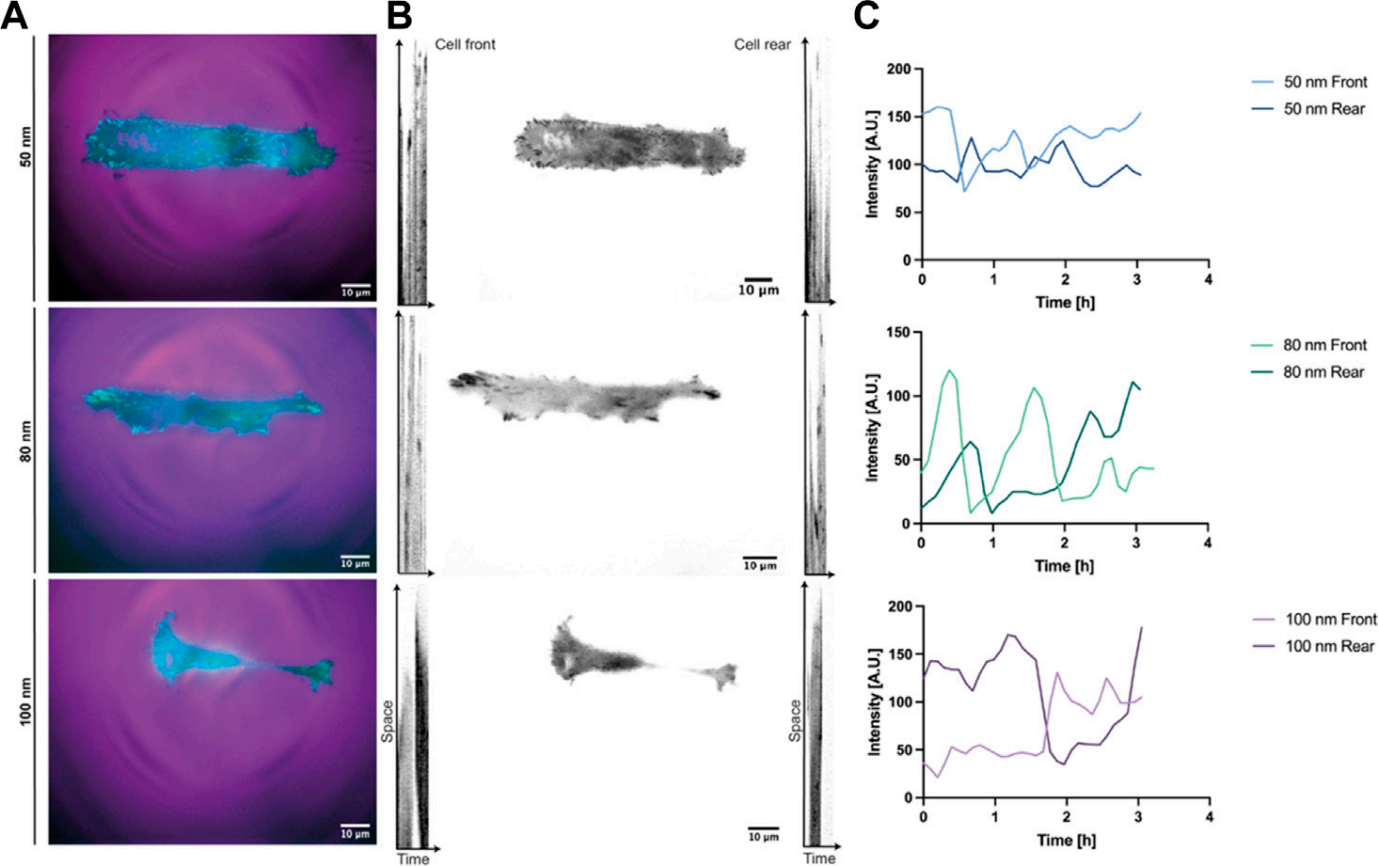
Reference kymographs of REF52-YFP-PAX cells migrating in 1D lines. Adhesion dynamics of REF52 cells stably expressing YFP-paxillin depicted in **(A)** cyan and in **(B)** black for all RGD-bound nanoparticle spacings. A cell front and rear positions were assigned according to the initial migrating direction. **(C)** Tracking of the initial cell front and rear representing sporadic direction changes during migration.

To further investigate the effect of ligand spatial distribution on adhesion, the dynamic evolution of paxillin-fused yellow fluorescent protein (YFP-PAX) was followed during migration for all ligand spacings (Figure 5, Supplementary Video S4). As discussed above, cells adhered to surfaces with 50 nm and 80 nm spacing established large adhesions to the substrate on both edges, presenting integrin clusters and forming mature focal adhesions (Supplementary Video S5). For a spacing of 100 nm, the adhesion on both sides of the cell was less stable showing few integrin clusters and focal adhesions (Figures 5A,B). The cell morphology at 100 nm was again more elongated with both ends stretching the cell body until adhesions on one side gave rise to a leading edge and an abrupt retraction at the rear end occurs. However, we initially thought that cells adhering to smaller ligand spacings were nonpolarized and that their transition to motile cells was due to subsequent adhesion losses after an adhesion competition of both ends (Supplementary Video S5). To our surprise, we observed that the polarization and subsequent directed migration can arise through continuous substrate nascent adhesions by the leading edge occupying the width of the line, while cycles of attachment and detachment to the substrate occur at the rear (Supplementary Video S4). Fluctuations in cell-substrate adhesions have been reported to induce the transition from a symmetrically extended cell without polarizing to a motile cell. When the adhesion force is higher, the adhesions at the back of the cell are more stable. This causes higher friction when the front of the cell exerts traction forces, resulting in a slower migration (Ron et al., 2020). These migratory adhesion dynamics have also been observed in 1D mesenchymal migration with a uniform matrix coating, where a cluster growth at the leading edge and smaller adhesion areas at the trailing edge were observed (Hennig et al., 2020), corroborating once more that adhesion strength to the substrate plays a main role in cell morphology, motility and speed for directed migration. For each stick-slip cycle observed, the length of the cells was recovered and the direction of migration could sporadically change, as shown in Figure 5C, where the preset front and back positions changed due to a possible repolarization. These events were mostly observed for larger ligand spacings or until the cell encountered another cell while migrating in the adhesive tracks (supplementary video 4–80 nm spacing).

**FIGURE 6:**
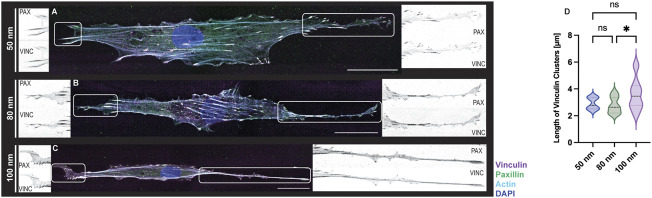
FA formations of REF-YFP-PAX cells adhered on 1D micro-nanopatterned striped surfaces. Immunofluorescence images with labeled vinculin (magenta), paxillin (green), F-actin (cyan), and nuclei (blue) were obtained for RGD-functionalized gold nanoparticles **(A–C)**. For all gold nanoparticle spacings, close-up views of paxillin (PAX) and vinculin (VINC) clusters observed at both ends of each cell are shown in black and white. Scale bars, 30 μm. **(D)** Violin plot depicting the size distribution of vinculin clusters for functionalized gold nanoparticles (*n* = 15 cells per gold nanoparticle spacing; total *n* = 45 cells). A one-way ANOVA test with a post hoc Tukey’s multiple comparison test, *ns* not significant, *p < 0.05.

Lastly, REF52 cells expressing YFP-paxillin were fixed after time-lapse imaging and stained for vinculin and F-actin to visualized FA formation. (Figure 6). Images of immunostained cells adhering to the biofunctionalized micro-nanopatterned surfaces were acquired in a set of 2–3 image tiles, as the front and rear of the cells were widely separated. The images were connected by pairwise stitching to form a complete image (Preibisch et al., 2009) for subsequent FA analysis. As expected from previous observations, cells exhibited an elongated cytoskeletal structure with actin stress fibers along the micropatterned lines with increasing ligand spacing (Figures 6A–C). However, actin stress fibers were more defined and stretched with decreasing ligand spacing, suggesting that cell tension decreases when ligand spacing is further apart since this is regulated by the formation of focal adhesions, as demonstrated earlier (Selhuber-Unkel et al., 2010). As shown in Supplementary Videos S4, S5, a mobile cell requires continuous and coordinated formation of FAs at the leading edge and disengagement at the rear to generate directional migration. Although this cannot be seen on fixed images, the colocalization of the adaptor proteins, vinculin and paxillin, demonstrates that the focal adhesions are stable at the leading edge of the cells, while the trailing edge is observed to be more elongated with fewer focal adhesions (Figures 6A–C insets). Furthermore, the quantifications of the length of the focal adhesions showed a uniform distribution with smaller ligand spacings while a greater variation was shown for 100 nm (Figure 6D). Once again, these results agree with previous work where stable cell adhesion was observed when the distance between the functionalized gold nanoparticles is less than 73 nm, showing a strong presence of focal adhesions around the nucleus and along the cell extension. However, in control samples, an undefined distribution of vinculin was reported in addition to disorganized actin filaments throughout the cell body (Arnold et al., 2004).

## Discussion

In this work, we show the integration of two well-established techniques to develop micro-nanopatterned substrates to generate a hierarchical platform for the regulation of cell adhesion and migration in 1D as a function of integrin clustering. Maskless photolithography technique enabled the patterning of micron-wide linear tracks on surfaces with spaced gold nanoparticles decorated with adhesive molecules to study mesenchymal adhesion and migration at the molecular scale (Figure 1). This photopatterning method relies on spatially controlled UV light exposure and standard photolithography methods to create 1D adhesive tracks. This was possible by employing a now commercially available wide-field photolithographic system where negative resist-coated nanopatterned substrates were held in a motorized stage and exposed to UV projecting the predesigned pattern. Unlike previous work that used photomasks to create gaps between passivated surfaces for further coating with an ECM ligand (Fink et al., 2007), this is a non-contact method enabling the preservation of the nanoarray for further biofunctionalization. In addition, it is a method that reduces manufacturing cost and time, since the patterns are designed *via* open-source software and it takes seconds to a few minutes to project a pattern onto the surface, similarly to current direct writing methods, such as laser protein patterning (Scott et al., 2012). The BCMNL technique, developed almost 20 years ago, has been an essential tool in the field of mechanobiology, as it is one of the first bottom-up approaches to create adhesive nanopatterns in a spatially controlled manner. This technique based on self-assembled polymeric systems has advantages over other nanopatterning techniques, such as dip-pen nanolithography (DPN) (Piner et al., 1999) and microcontact printing (µCP) (Qin et al., 2010), since the polymer molecules can be positioned at a nanometric level by means of spin (Denis et al., 2002) or dip coating the substrates into the micellar solution without the need of high-resolution microscopy probes or stamps. By combining conventional top-down and bottom-up strategies, it is possible to partially mimic the cellular heterogeneous external environment in 1D which is closely related to cell migration in 3D (Doyle et al., 2009).

We were able to extract morphological parameters and identify the migration behavior of REF-YFP-PAX fibroblasts attached to 1D linear patterns with RGD-bound gold nanoparticles specific for integrin αvβ3 (Cavalcanti-Adam et al., 2007). We found that within a uniform cell population, a variety of spontaneous migratory behaviors were exhibited by single mesenchymal cells in the absence of external cues. The generalized migration behavior for the three ligand spacings, 50–80–100 nm, is explained by the generic stick-slip mechanism in which there is an initial symmetrical elongation with a spontaneous symmetry breaking generated by the loss of adhesions at one end and migration to the opposite side (Figure 3). This mechanism depends on the dynamics of focal adhesions and contractile forces without an established polarity (Hennig et al., 2020). Although the fabrication of the surfaces is highly reproducible, we find variability in cellular migratory behavior on surfaces with lower ligand spacings. According to our observations, there is a dependency between cell length and adhesion strength. On substrates with ligand spacings of 50 nm, a lower displacement and migration rate may be due to the adhesion force that exists at both ends of the cell pulling the cell (Supplementary Video S5) until one side loses the adhesions and the rear retracts while exerting a large friction. Changes in these adhesions can bring an immobile cell into a migratory state. On the contrary, with ligand spacing of 100 nm, the adhesion strength is lower at the ends, presenting fewer focal adhesions, which leads to a higher rate of cell migration and displacement due to its high instability, experiencing more cycles of protrusion-retraction (Figure 4, Supplementary Video S4). This dependency on cell length and adhesion force has already been described previously in order to explain the different migration patterns within the stick-slip mechanism in uniformly ECM-coated 1D linear patterns (Ron et al., 2020).

This explanation may seem contradictory since the present method is based on an adhesion mediated by integrins and according to the catch-bond model, by reducing the spacing between ECM ligands, the force will decrease and cell movement will be facilitated, and *vice versa* when increasing the spacing between ligands (Oria et al., 2017). However, it is also known that in rigid substrates the adhesion force will increase, thus stabilizing focal adhesions (Liu et al., 2016). It has been reported that the size of these complexes can normally be in the range of 1–5 μm, although they can be larger than 5 µm. If the focal adhesions are less than 1 μm, then the cell can move nevertheless without stability or tension in the cytoskeleton. In contrast, if the size is greater than 1 μm, then medium and long adhesions can help in cell stabilization during migration and can develop into fibrillar or central adhesions that help remodel cell shape (Biggs et al., 2009; Luo et al., 2022). This supports the quantifications obtained for the cells adhered to the biofunctionalized substrates and controls (Figure 6D) which correlate with the cellular shapes we observed after performing the immunostaining (Figure 6) although great variability in FAs size for 100 nm ligand spacing was observed.

To conclude, this work offers a new flexible platform to create micro-nanopatterned substrates to generate biomaterial structuring at different length scale. The variation in the spacing of gold nanoparticles and their functionalization with specific ligands in delimited regions of the substrate can help in understanding the molecular process of cell-matrix mechanosensing and the following biochemical signals that lead to cell polarization and migration. Especially for integrin-based migration, carried out through the recruitment of multiprotein structures, is orchestrated sequentially and is also reversible. However, this work also leaves open questions, since the crosstalk between adaptor proteins within the integrin-ECM complex and downstream events are still fragmented. Further studies at various scales and times will be essential to elucidate the intrinsic mechanism involved in directed cell migration and protein interactions during cell-matrix processes in individual cells. Future studies will extend this to collective cell behavior in order to understand physiological and pathological processes at larger biological scales.

## Data Availability

The raw data supporting the conclusion of this article will be made available by the authors, without undue reservation.
